# Examining the Associations between Walk Score, Perceived Built Environment, and Physical Activity Behaviors among Women Participating in a Community-Randomized Lifestyle Change Intervention Trial: Strong Hearts, Healthy Communities

**DOI:** 10.3390/ijerph16050849

**Published:** 2019-03-08

**Authors:** Brian K. Lo, Meredith L. Graham, Sara C. Folta, Lynn C. Paul, David Strogatz, Miriam E. Nelson, Stephen A. Parry, Michelle E. Carfagno, David Wing, Michael Higgins, Rebecca A. Seguin

**Affiliations:** 1 Division of Nutritional Sciences, Cornell University, Ithaca, NY 14853, USA; bl592@cornell.edu (B.K.L.); mlg22@cornell.edu (M.L.G.); sp2332@cornell.edu (S.A.P.); mec329@cornell.edu (M.E.C.); 2 Friedman School of Nutrition Science and Policy, Tufts University, Boston, MA 02111, USA; sara.folta@tufts.edu; 3 College of Education, Health and Human Development, Montana State University, Bozeman, MT 59717, USA; lpaul@montana.edu; 4 Bassett Healthcare Network, Cooperstown, NY 13326, USA; david.strogatz@bassett.org; 5 Hampshire College, Amherst, MA 01002, USA; miriamnelson@hampshire.edu; 6 Exercise and Physical Activity Resource Center, University of California, San Diego, CA 92093, USA; dwing@eng.ucsd.edu (D.W.); mdhiggins@eng.ucsd.edu (M.H.)

**Keywords:** built environment, physical activity, Walk Score, obesity, rural health, intervention

## Abstract

Little is known about the relationship between perceived and objective measures of the built environment and physical activity behavior among rural populations. Within the context of a lifestyle-change intervention trial for rural women, Strong Hearts, Healthy Communities (SHHC), we examined: (1) if Walk Score (WS), an objective built environment measure, was associated with perceived built environment (PBE); (2) if WS and PBE were associated with moderate-to-vigorous physical activity (MVPA); and (3) if MVPA changes were modified by WS and/or PBE. Accelerometers and questionnaires were used to collect MVPA and PBE. Bivariate analyses and linear mixed models were used for statistical analyses. We found that WS was positively associated with perceived proximity to destinations (*p* < 0.001) and street shoulder availability (*p* = 0.001). MVPA was generally not associated with WS or PBE. Compared to controls, intervention group participants increased MVPA if they lived in communities with the lowest WS (WS = 0), fewer perceived walkable destinations, or extremely safe perceived traffic (all *p* < 0.05). Findings suggest that WS appears to be a relevant indicator of walkable amenities in rural towns; results also suggest that the SHHC intervention likely helped rural women with the greatest dearth of built environment assets to improve MVPA.

## 1. Introduction

Although maintaining certain levels of moderate-to-vigorous physical activity (MVPA) is crucial to chronic disease control and prevention, more than half of Americans do not meet the current physical activity recommendations [1]. Rural populations in particular are less likely to perform adequate MVPA compared to their non-rural counterparts [2,3,4]. Such geographic disparities could be due to rural communities’ lack of access to recreational facilities as well as geographic and topographic features that inhibit active living [5]. Furthermore, rural women report more caregiving duties [6], less social support [6], and fewer role models for physical activity [7]. These may exacerbate barriers to physical activity in this population.

Over the past few decades, considerable effort has been made to measure and characterize the built environment to better understand the impacts of the built environment on human behaviors and health [8]. One commonly used tool to objectively characterize the built environment is Walk Score [9]. Walk Score is a private company that is now part of Redfin, and its stated mission is “To promote walkable neighborhoods” [10]. In general, Walk Score was created by a multidisciplinary advisory board, and the score is tabulated based on three components: (1) the distance to a group of destinations and amenities (e.g., restaurants, shopping, schools, green spaces); (2) the population density; and (3) the road metrics (e.g., block length and the intersection density) of a given address. Data sources include Google, Education.com, Open Street Map, the U.S. Census, Localeze, and places added by the Walk Score user community. Walk Score ranges from 0 and 100, with higher scores indicating higher levels of walkability. Walk Score has been established as a valid measure in estimating accessibility to nearby amenities in some urban neighborhoods [11,12], and, in a few instances, it has been associated with physical activity and health [13,14,15]. Because Walk Score is freely accessible online (http://www.walkscore.com/), it has emerged as a commonly utilized tool to characterize neighborhoods’ built environment among researchers and the general public [9]. However, a major limitation of Walk Score is that it does not account for other characteristics of the built environment, such as physical activity resources, aesthetics, and safety, which are often perceived as important influences on physical activity engagement among rural adults [16,17,18,19,20].

Given that rural communities are dispersed over a large geographical area and have limited transportation infrastructure, it is time- and labor-intensive and costly to objectively capture the built environment features of rural communities. Walk Score has the potential to serve as an alternative objective built environment proxy. However, the extent to which Walk Score and resident perceptions of the built environment capture the same construct is unclear; it is also unclear whether they can be used interchangeably in the rural context. Prior studies conducted in urban areas showed mixed results regarding the associations between Walk Score and the perceived built environment. For example, Carr, Dunsiger, and Marcus found that Walk Score was associated with a group of Rhode Island residents’ perceived presence of sidewalks, street lights, and other pedestrians, as well as traffic and crime safety; however, they did not find any association between Walk Score and perceived access to community physical activity facilities [21]. Similarly, although Bereitschaft found that Walk Score was associated with perceived walkability among a group of residents in Nebraska, the strength of the association was stronger with suburban strip-mall corridors and weaker with recreational areas and small entertainment districts [22]. Given that rural communities often have a town center, but many residents live outside of the town center, it is unclear how those associations might vary in a rural context. Some have argued that perception is a better reflection of reality because it is a product of individuals’ experience over time [23]. Therefore, the first aim of the present study was to examine if Walk Score is associated with rural women’s perceived access to nearby amenities, perceived availability of physical activity resources, perceived aesthetics, and perceived safety. Findings will shed light on the potential for Walk Score to serve as an estimate of rural communities’ walkability.

While objective built environment measures such as Walk Score are often considered less biased than perceived built environment measures, a recent review found that perceived built environments are more strongly associated with physical activity than objectively measured built environment features [24]. However, most studies in this review were in urban and suburban settings [24], and the transferability of these findings to rural settings is unknown. Therefore, the second aim of the present study was to examine whether Walk Score and perceived built environment characteristics were associated with rural women’s MVPA. Since rural communities often face resource constraints in developing and implementing health promotion strategies [13], this would help to determine whether costly environmental interventions to improve the built environment are necessary or whether education about existing physical activity resources to improve awareness may be sufficient. Based on the findings of previous studies [24], we hypothesized that perceived built environment characteristics would be more strongly associated with rural women’s MVPA than Walk Score.

Moreover, although the built environment seems to influence physical activity, little research has explored how it may influence or modify the relationship between behavior change interventions with physical activity outcomes [25,26,27,28,29]. Recent reviews have suggested that future studies should examine the interaction between environmental attributes and physical activity to determine when, where, and for whom certain environmental features are important to consider [30,31,32]. Hence, the third aim was to examine if rural women’s MVPA changes were modified by their objective and/or perceived built environment within the context of a community-randomized lifestyle intervention trial: Strong Hearts, Healthy Communities (SHHC). Because SHHC was designed to improve MVPA regardless of the environmental conditions of the town, we hypothesized that there would be no significant interaction between Walk Score or perceived built environments and the intervention.

## 2. Materials and Methods 

### 2.1. Study Population and Setting

**Figure 1:**
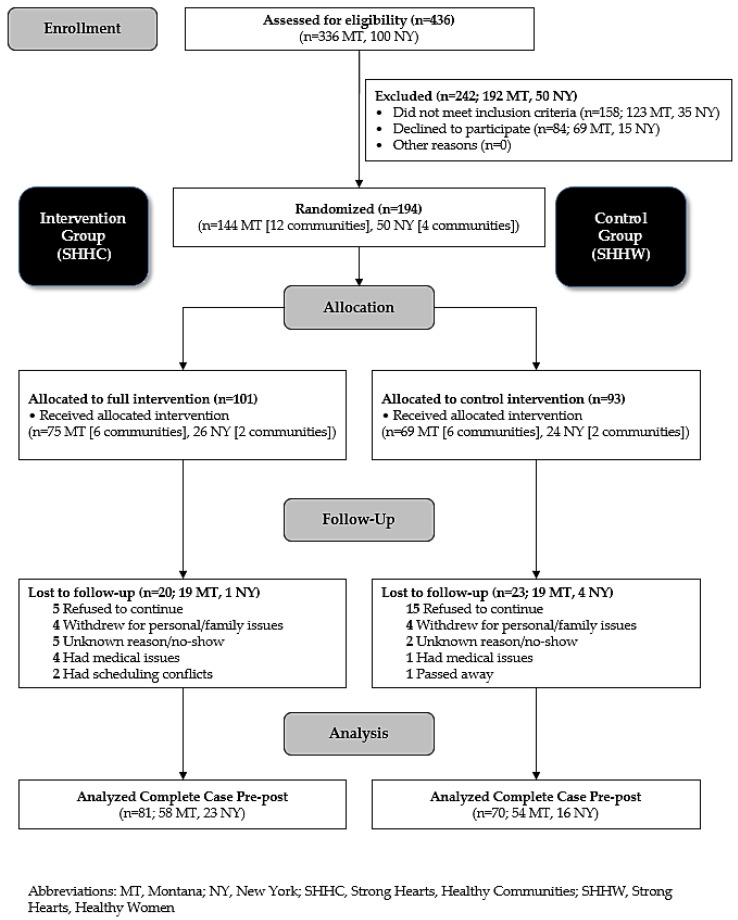
Study Flow: Strong Hearts, Healthy Communities, Montana and New York, 2015–2016.

The present study is a secondary analysis of the SHHC intervention trial. Study rationale, methods for recruitment, enrollment, data collection, and participant characteristics have been described elsewhere [33,34]. Briefly, 194 overweight, sedentary midlife and older women participated in a six-month community-randomized trial in 16 medically-underserved rural towns in Montana and New York between 2015 and 2016. Eight towns received the SHHC intervention (*n* = 101)—a 48-session (twice a week for 24 weeks) multilevel intervention focusing on improving physical activity and diet quality through in-class exercise sessions and skill-building activities, field-based learning, and other activities that foster supportive social and built environments for positive behavioral changes. The in-class exercise sessions included walking and aerobic dance DVDs or 20–30 min outdoor walks and progressive strength training using dumbbells. The other eight towns received a six-session (once a month for 24 weeks) education-only control intervention, Strong Hearts, Healthy Women (SHHW), that provided general information on healthy living (*n* = 93). The study flow diagram is shown in Figure 1.

All participants gave their informed consent for inclusion before they participated in the study. The study was conducted in accordance with the Declaration of Helsinki, and the protocol was approved by the Cornell University Institutional Review Board (Protocol #1402004505; approval date: 22 May 2015).

### 2.2. Measurements

#### 2.2.1. Demographics

Participants completed a questionnaire to provide demographic information at baseline [33,34].

#### 2.2.2. Objective Built Environment

The Walk Score of participants’ baseline home address was used to characterize the objective built environment (obtained at http://www.walkscore.com/). Walk Score uses a patented algorithm to measure the walkability of a given address based on the proximity to basic destinations including grocery, dining and drinking, shopping, parks, schools, errands, and culture and entertainment. After inputting an address on the website, a Walk Score ranging from 0 and 100 is issued. Briefly, points are given for amenities within 0.25 miles and points are deducted if amenities are distant. In addition, Walk Score captures pedestrian friendliness by accounting for population density and road metrics such as block length and intersection density. A Walk Score between 0 and 24 represents a community that is “Car-Dependent: almost all errands require a car”, between 25 and 49 is “Car-Dependent: most errands require a car”, between 50 and 69 is “Somewhat Walkable: some errands can be accomplished on foot”, between 70 and 89 is “Very Walkable: most errands can be accomplished on foot” and between 90 and 100 is “Walker’s Paradise: daily errands do not require a car”.

The Walk Score distribution of our sample was skewed to the lower end: 31.1% of participants had a Walk Score of 0, and 29.2% of the participants had a Walk Score between 1 and 24 (see Table 1). Therefore, we divided the lowest Walk Score level into two categories “Walk Score of 0” and “Walk Score 1–24.” Furthermore, since only two participants had Walk Scores greater than 69, we combined them with the “Walk Score 50–69” category. This highest category is referred to as “Walk Score ≥ 50” in this paper.

#### 2.2.3. Perceived Built Environment

Participants’ perceptions of their community’s built environment were assessed using the questionnaire developed by Boehmer et al. [35]. Community was defined as the area where participants live.

On a four-point Likert scale (i.e., strongly agree to strongly disagree), participants reported whether there were many destinations (e.g., a store, a workplace, a place of worship) to go within easy walking distance from their home (perceived proximity to destinations); there were sidewalks on most of the streets in their community (perceived sidewalk availability); there were shoulders on the streets that allowed for safe walking or biking (perceived street shoulder availability); there were bike lanes on most of the streets in their community (perceived bike lane availability); there were many places to be physically active in their community not including streets for walking or jogging (perceived physical activity facility availability); there was equipment available for physical activity in their community (perceived physical activity equipment availability); there were many interesting things to look at while walking in their community (perceived landscape diversity); there were trees along the streets in their community (perceived greenery); their community was well-maintained (perceived maintenance); and their community was generally free from garbage, litter, or broken glass (perceived cleanliness).

In addition, participants also reported their perceptions about both crime and traffic safety related to walking and biking in their community on a four-point Likert scale (i.e., extremely safe to not at all safe).

Some of the data had a non-normal distribution. Therefore, all of the perceived variables were recoded to allow meaningful interpretation. “Strongly agree” and “agree” responses were collapsed into “agree”, and “strongly disagree” and “disagree” responses were collapsed into “disagree”. “Slightly safe” and “not at all safe” responses were collapsed into “unsafe”. This same approach was used in the original Boehmer et al. article [35]. 

#### 2.2.4. Physical Activity

ActiGraph Model GT3X+ and GT3X-BT accelerometers (ActiGraph LLC, Pensacola, FL, USA) were used to measure participants’ average MVPA daily minutes and the percentage of wear time spent in MVPA (% MVPA) at baseline and post-intervention (24 weeks). The models are completely compatible and gather identical data in terms of accelerations (raw data) and counts (processed data). Participants were asked to wear the accelerometer at the hip for seven days and remove it when sleeping, bathing, and swimming. In addition, participants were given a daily log sheet to record their wear and non-wear periods. Accelerometers were set to gather raw data at 30 Hz; raw data files were aggregated to 60-s epoch length for analysis. Firmware version 3.2.1 (initially released March 2013, ActiGraph, LLC, Pensacola, FL, USA) was used for GT3X+ devices, and a combination of version 1.6.0 (December 2015) and 1.7.0 (March 2016) were used for GT3X-BT. 

In line with currently accepted best practice [36], the Low Frequency Extension (LFE) filter was used when processing the raw data to counts for minute-level analysis. The algorithm developed by Choi et al. [37] was then used to identify (and exclude) non-wear time via vertical axis counts. Specifically, non-wear was defined as ≥90 min of consecutive zero counts and a spike tolerance of 2 min with a 30-min window of zero counts upstream and downstream of each observed spike. An observation day was considered valid if there was greater than or equal to 600 min (i.e., 10 h) of valid wear time. Participants were included for analysis if they had a minimum of five valid days, or four valid days totaling a minimum of 3000 min of wear. These inclusion criteria, both at the daily and participant level, have been used in other studies, most notably the U.S. National Health and Nutrition Examination Survey (NHANES) accelerometer analyses [38,39]. Because participants were essentially healthy and without disability, Freedson cut-points were used to determine minute-level intensity of physical activity [40].

### 2.3. Statistical Analysis

Descriptive statistics were used to characterize study participants. We used *t*-tests and Kruskal–Wallis tests to examine demographic differences between intervention and control groups at baseline across continuous variables, normally distributed variables, and non-normally distributed variables, respectively. Chi-square tests and Fisher’s exact tests were used to examine differences between intervention and control groups at baseline across categorical variables depending on small cell counts.

Bivariate analysis utilizing Fisher’s exact tests, suitable for analyzing small cell sizes, were conducted to examine the associations between Walk Score and participants’ perceived built environment characteristics at baseline.

Linear mixed models were used to examine Walk Score and perceived built environment characteristic associations with baseline MVPA (both MVPA minutes per day and % MVPA). Models controlled for participants’ age, marital status, and education. Study site was treated as a random effect. Because baseline MPVA values were skewed to the right, square root transformation was performed for baseline MVPA variables to meet model assumptions (baseline median MVPA minutes per day was 10.3 minutes (Interquartile range (IQR) = 14.6) and baseline median % MVPA was 1.3% (IQR = 1.7%)).

Based on our previous findings [41], which showed that both MVPA minutes per day and % MVPA increased in SHHC participants, linear mixed models were used to examine whether the intervention’s effects on physical activity changes were modified by Walk Score and baseline perceived built environment characteristics. In each model, an interaction term was used between intervention group and the built environment variable. Models controlled for study site, participants’ age, education, marital status, and baseline physical activity level.

A Bonferroni correction was applied to account for multiple between-group comparisons within each model. Model assumptions were checked and met.

Missing data were checked using the Little’s missing completely at random (MCAR) test [42], and it confirmed that our missing data were MCAR (*p* = 0.454); therefore, we did not conduct further imputation models.

Analyses were conducted using SPSS 25.0 (SPSS Inc., Chicago, IL, USA). The type I error rate was set at 0.05.

## 3. Results

### 3.1. Participant Characteristics at Baseline

Details of study participants’ demographics are outlined in Table 1; additional details have been reported elsewhere [33]. There were no demographic differences between intervention and control groups at baseline. In addition, physical activity levels were similar at baseline between groups. In terms of built environment characteristics (Table 1), there were differences between groups at baseline: compared to participants in the intervention group, those in the control group had a higher mean Walk Score for their home location (*p* = 0.001) and more participants reported closer proximity to destinations (*p* = 0.006), greater availability of sidewalks (*p* = 0.020), and greater availability of physical activity equipment (*p* = 0.007); in contrast, participants in the intervention group perceived more greenery in their community than controls (*p* = 0.027).

### 3.2. Relationships between Walk Score and Perceived Built Environment Characteristics

Walk Scores were associated with perceived proximity to destinations and perceived street shoulder availability. Participants with higher Walk Scores were more likely to perceive being close to destinations (*p* < 0.001) and having street shoulders (*p* = 0.001) in their community. Walk Scores were marginally associated with perceived sidewalk availability (*p* = 0.060). Walk Scores were not associated with other perceived physical activity resource availability (e.g., bike lanes, physical activity facilities, physical activity equipment) or any of the perceived aesthetic or safety characteristics (Table 2).

### 3.3. Associations of Walk Score and Perceived Built Environment Characteristics with Baseline MVPA

Only perceived cleanliness was positively associated with participants’ baseline average MVPA minutes per day (β = 0.80, 95% CI = 0.16–1.43, *p* = 0.015) and % MVPA (β = 0.28, 95% CI = 0.06–0.50, *p* = 0.012). Our supplementary analyses found that perceived cleanliness was also associated with perceived sidewalk availability, perceived maintenance, and perceived crime and traffic safety (all *p* < 0.05). Supplementary Table S1 shows Goodman and Kruskal’s gamma correlations among all perceived built environment variables.

Walk Scores and the other 11 perceived built environment characteristics were not associated with participants’ baseline MVPA.

### 3.4. Built Environment and Intervention Effects on Physical Activity

None of the interaction terms between intervention group and built environment measures (both Walk Score and perceived built environment characteristics) were statistically significant.

However, between-group comparisons revealed that among those who lived in communities with a Walk Score of zero, women in the intervention group increased average MVPA minutes per day (between-group difference: Δ = 12.7, 95% CI = 1.4–24.0, *p* = 0.028) and % MVPA (between-group difference: Δ = 1.6, 95% CI = 0.2–2.9, *p* = 0.023) compared to controls (Table 3). Similarly, among those who perceived living far from walkable destinations, women in the intervention group increased % MVPA compared to controls (between-group difference: Δ = 1.0, 95% CI = 0.003–2.0, *p* = 0.049) (Table 4).

In addition, among those who lived in communities that were perceived having extremely safe traffic, women in the intervention group increased average MVPA minutes per day compared to controls (between-group difference: Δ = 14.8, 95% CI = 0.9–28.8, *p* = 0.038) (Table 4).

## 4. Discussion

In the present study, we found that Walk Scores were associated with perceived proximity to destinations and perceived street shoulder availability and were marginally associated with perceived sidewalk availability among rural women. In addition, baseline MVPA was generally not associated with built environment perceptions or Walk Score. Finally, changes in intervention participants’ MVPA were enhanced if they lived in a place with a lower Walk Score, fewer perceived walkable destinations, or with safer perceived traffic.

Similar to other studies [43], Walk Score and perceived built environments seem to be concordant on the underlying construct of number and density of walkable amenities; these objective and perceived constructs might therefore be used interchangeably, even in the rural context. However, Walk Score did not correlate with other perceived built environment characteristics related to perceived physical activity resources, aesthetics, or safety. These findings support previous suggestions that future studies should use other supplementary measures (both objective and perceived) to characterize built environment features that are not addressed by Walk Score, such as landscape diversity, greenery, maintenance, cleanliness, and crime [21].

To our knowledge, no previous studies have examined the association between Walk Score, perceived built environment characteristics, and MVPA among sedentary, overweight or obese, midlife and older women living in rural communities in the U.S. Among the few studies conducted among other rural adult populations in the U.S., findings on the associations between built environment characteristics and physical activity were mixed [44,45]. In general, aesthetics; safety from crime; and presence of trails, parks, or recreational activities were more consistently associated with physical activity among rural adults [46]. In our study, we only found an association between perceived community cleanliness and physical activity, and perceived cleanliness was associated with perceived sidewalk availability, perceived maintenance, and perceived crime and traffic safety. However, findings between perceived cleanliness and MVPA in our study could be due to the high proportion of participants perceiving their community as clean rather than an actual association between physical activity and community cleanliness.

There are a few potential explanations for the general lack of association between built environment characteristics and MVPA in our study. First, the survey instrument used in this study might have missed other unique aspects of the built environment related to physical activity in rural areas. In particular, the majority of our participants perceived far proximity to destinations, no bike lanes, aesthetically pleasing outdoor space, and low crime rates in their community. The lack of heterogeneity in these responses hindered us from better understanding whether variations of these built environment features would impact rural residents’ physical activity differently. Additionally, through our previous formative work for the SHHC intervention, we learned that midlife and older rural residents are more interested in nature-based activities such as hiking, hunting, and fishing [47]. Therefore, proximity to destinations or active transportation opportunities might be less relevant to their physical activity. One recent Australian study that explored the salience of urban physical activity environment constructs among rural adults also found that some built environment features were less relevant to rural physical activity, including personal safety related to crime, availability of walkable destinations, and aesthetics [48]. These are important discoveries, and similar explorations are needed in the U.S. so that researchers can appropriately quantify rural built environment features related to physical activity in future studies. However, in the present study, we wanted to use the data that we collected to see if we could shed further light on how built environment perceptions and a publicly available objective measure (i.e., Walk Score) may align. Second, due to the design and eligibility criteria of our intervention trial, participants were sedentary at baseline, which limited the variability of MVPA in our analyses. Associations between baseline MVPA and the built environment are specific to our sample of overweight and obese rural women that enrolled in a randomized trial. Third, physical activity is a broad construct that includes leisure physical activity, household physical activity, occupational activity, and transportation-related physical activity [49]. It is likely that women in our study perform these various types of activities outside of their community, especially for those who travel outside of their community for work and leisure activities. Fourth, physical activity is an interplay between individual, social, and environmental factors [50]. Physical activity and how it is associated with the built environment may be moderated by the individual and social factors that are relevant to engaging in physical activity, such as self-efficacy, social support, and health status [51]. For instance, since women are more likely to engage in physical activity with others [52], the association between engaging in physical activity for women may be with the social environment rather than the built environment. Future studies should examine where rural populations actually perform different types of physical activity and how various individual, social, and environmental factors influence their physical activity behaviors.

Furthermore, our findings related to Walk Score and perceived proximity to destinations’ effects on participants’ MVPA changes contradicted previous hypotheses suggesting that behavior change interventions would be more effective in better-resourced communities and communities that have fewer barriers related to physical activity [53]. In medically underserved rural communities, those living in communities with fewer walkable destinations as measured by Walk Score and perceived proximity to destinations appeared to achieve superior benefit from the SHHC intervention in increasing MVPA. Similar to our study, one study in rural southeastern North Carolina found that among adults who participated in a lifestyle intervention study, those who lived further from gyms and in areas with a lower density of gyms had greater increases in physical activity and walking steps than those who lived in communities with close proximity to amenities and destinations [27]. Similarly, Kerr et al. found that men in a lifestyle intervention, but not women, improved their walking more if they lived in less-walkable communities in suburban San Diego [28]. In contrast, Zenk et. al did not find any differential effects due to environmental characteristics after implementing a walking intervention program in Chicago with African American women [29]. These differences could be due to the differences in geography, nature of the intervention, and variety in measurement instruments. Our findings suggest that a lifestyle intervention in the least-walkable communities increased physical activity and holds promise for the intervention across medically underserved rural and isolated communities.

Our findings also suggest a potential ceiling effect such that those who live in communities with nearby destinations did not increase their MVPA further after participating in the SHHC intervention. In contrast, those who live in communities with fewer amenities overcame some environmental barriers to increase their MVPA through the SHHC intervention, which provided an indoor space for exercise. These findings suggest that researchers and practitioners should consider selecting communities that have fewer walkable destinations to implement the SHHC program since that is where MVPA improvements seemed to have been maximized. Further examination through in-depth interviews and focus groups is needed to understand what enabled participants living in communities with fewer amenities to overcome the environmental barriers to exercise more.

Lastly, our analyses also found that participants who live in communities with extremely safe traffic benefited more from the SHHC intervention in increasing MVPA. These findings are supported by other studies that found poor traffic safety was a barrier to physical activity in rural communities [54]. It could be that with fewer traffic safety concerns, midlife and older adults feel safer to engage in physical activity in their community without fear of the unexpected.

There were several limitations in our study. First, our participants were not purposively recruited based on their communities’ built environment characteristics. Future studies should use a bigger sample and include purposeful variation in built environment features and amount of physical activity to further understand the interplay between rural built environment and physical activity. Second, it is possible that environmental changes may have occurred in the time period between participants’ perception data collection (2015) and Walk Score calculations (2017). Third, we did not differentiate between different types of physical activity. Future studies should focus on specific physical activity domains to better understand the relationships between the built environment and specific types of physical activity. Fourth, our participants were sedentary, overweight or obese, midlife and older women living in medically underserved rural communities. Findings may not be generalizable to other populations. Fifth, since participants were asked to participate in a lifestyle change intervention trial, we might have recruited a group of individuals that were eager to measure and improve their physical activity level. Finally, the present study is a secondary data analysis of the larger SHHC intervention that was not powered to test the interactions between intervention groups and built environment features. Future studies need to use a larger sample size to obtain sufficient statistical power to detect meaningful moderation effects.

## 5. Conclusions

Findings from the present study contribute to the limited research in this area. While Walk Score appears to match rural residents’ perception of the availability of nearby amenities, physical activity was not associated with local built environment characteristics. Our additional analyses suggest that a community-based lifestyle intervention helped rural overweight and obese women living in communities with less walkable destinations to overcome environmental barriers to physical activity. Our results warrant more research to understand the complex relationships between built environment and physical activity among rural adults. Evaluating the environmental conditions that maximize the effects of physical activity interventions would also help researchers and practitioners better use rural communities’ resources. 

## Figures and Tables

**Table 1:** Baseline characteristics of participants, Walk Score, and perceived built environment characteristics.

Characteristics	Total	Intervention	Control	*p*-Value
Age, mean (SD) (Total *n* = 194; Intervention *n* = 101; Control *n* = 93)	58.9 (9.5)	59.0 (9.5)	58.7 (9.7)	0.834 ^a^
Relationship status, *n* (%)				
In a relationship	132 (71.4)	70 (73.7)	62 (68.9)	0.471 ^b^
Not in a relationship	53 (28.6)	25 (26.3)	28 (31.1)	
Education level, *n* (%)				
High school or less	42 (22.8)	22 (23.4)	20 (22.2)	
Technical or vocational school/some college	55 (29.9)	30 (31.9)	25 (27.8)	
College graduate	58 (31.5)	28 (29.8)	30 (33.3)	0.904 ^b^
Postgrad/professional	29 (15.8)	14 (14.9)	15 (16.7)	
BMI, mean (SD) (Total *n* = 194; Intervention *n* = 101; Control *n* = 93)	35.2 (6.5)	34.9 (6.1)	35.5 (6.8)	0.532 ^a^
Number of chronic diseases, mean (SD) (Total *n* = 186; Intervention *n* = 96; Control *n* = 90)	1.8 (1.5)	2.0 (1.7)	1.7 (1.4)	0.320 ^c^
MVPA min/day, mean (SD) (Total *n* = 183; Intervention *n* = 95; Control *n* = 88)	14.3 (13.1)	14.9 (14.0)	13.7 (12.1)	0.643 ^c^
% MVPA (SD) (Total *n* = 183; Intervention *n* = 95; Control *n* = 88)	1.7 (1.5)	1.8 (1.6)	1.6 (1.5)	0.663 ^c^
Walk Score, mean (SD) (Total *n* = 161; Intervention *n* = 82; Control *n* = 79)	21.0 (21.8)	15.2 (18.2)	27.1 (23.6)	**0.001** ^c^
Walk Score levels, *n* (%)				
0	50 (31.1)	33 (40.2)	17 (21.5)	
1–24	47 (29.2)	24 (29.3)	23 (29.1)	**0.004** ^b^
25–49	40 (24.8)	20 (24.4)	20 (25.3)	
≥50	24 (14.9)	5 (6.1)	19 (24.1)	
Perceived close proximity to destinations, *n* (%)				
Agree	87 (46.8)	35 (36.8)	52 (57.1)	**0.006** ^b^
Disagree	99 (53.2)	60 (63.2)	39 (42.9)	
Perceived sidewalk availability, *n* (%)				
Agree	120 (64.2)	54 (56.3)	66 (72.5)	**0.020** ^b^
Disagree	67 (35.8)	42 (43.8)	25 (27.5)	
Perceived street shoulder availability, *n* (%)				
Agree	97 (52.2)	47 (49)	50 (55.6)	0.368 ^b^
Disagree	89 (47.8)	49 (51)	40 (44.4)	
Perceived bike lane availability, *n* (%)				
Agree	8 (4.3)	5 (5.2)	3 (3.3)	0.721 ^d^
Disagree	179 (95.7)	91 (94.8)	88 (96.7)	
Perceived physical activity facility availability, *n* (%)				
Agree	97 (51.9)	47 (49)	50 (54.9)	0.413 ^b^
Disagree	90 (48.1)	49 (51)	41 (45.1)	
Perceived physical activity equipment availability, *n* (%)				
Agree	113 (60.4)	49 (51)	64 (70.3)	**0.007** ^b^
Disagree	74 (39.6)	47 (49)	27 (29.7)	
Perceived landscape diversity, *n* (%)				
Agree	126 (67.4)	67 (69.8)	59 (64.8)	0.470 ^b^
Disagree	61 (32.6)	29 (30.2)	32 (35.2)	
Perceived greenery, *n* (%)				
Agree	166 (88.8)	90 (93.8)	76 (83.5)	**0.027** ^b^
Disagree	21 (11.2)	6 (6.3)	15 (16.5)	
Perceived maintenance, *n* (%)				
Agree	140 (74.9)	71 (74)	69 (75.8)	0.769 ^b^
Disagree	47 (25.1)	25 (26)	22 (24.2)	
Perceived cleanliness, *n* (%)				
Agree	161 (86.1)	82 (85.4)	79 (86.8)	0.783 ^b^
Disagree	26 (13.9)	14 (14.6)	12 (13.3)	
Perceived crime safety, *n* (%)				
Extremely safe	63 (33.7)	32 (33.3)	31 (34.1)	0.807 ^b^
Quite safe	104 (55.6)	55 (57.3)	49 (53.8)	
Unsafe	20 (10.7)	9 (9.4)	11 (12.1)	
Perceived traffic safety, *n* (%)				
Extremely safe	32 (17.1)	16 (16.7)	16 (17.6)	0.949 ^b^
Quite safe	105 (56.1)	55 (57.3)	50 (54.9)	
Unsafe	50 (26.7)	25 (26)	25 (27.5)	

^a^*t*-test; ^b^ Chi square test; ^c^ Kruskal–Wallis test; ^d^ Fisher’s Exact Test; Significant *p*-values are indicated in bold. BMI: body mass index; MVPA: moderate-to-vigorous physical activity.

**Table 2:** Bivariate associations between Walk Score and perceived built environment characteristics among participants at baseline.

Built Environment Perceptions	Walk Score = 0 (*n* = 50)		Walk Score 1–24 (*n* = 47)		Walk Score 25–49 (*n* = 40)		Walk Score ≥50 (*n* = 24)		*p*-Value
	No.	%	No.	%	No.	%	No.	%	
Perceived close proximity to destinations									
Agree	2	4.0	22	46.8	31	77.5	20	87.0	**<0.001**
Disagree	48	96.0	25	53.2	9	22.5	3	13.0	
Perceived sidewalk availability									
Agree	27	54.0	29	61.7	29	72.5	20	83.3	0.060
Disagree	23	46.0	18	38.3	11	27.5	4	16.7	
Perceived street shoulder availability									
Agree	19	38.8	20	42.6	31	77.5	14	58.3	**0.001**
Disagree	30	61.2	27	57.5	9	22.5	10	41.7	
Perceived bike lane availability									
Agree	3	6.0	0	0.00	1	2.5	1	4.2	0.330
Disagree	47	94.0	47	100.0	39	97.5	23	95.8	
Perceived physical activity facility availability									
Agree	25	50.0	20	42.6	24	60.0	14	58.3	0.370
Disagree	25	50.0	27	57.4	16	40..0	10	41.7	
Perceived physical activity equipment availability									
Agree	26	52.0	28	59.6	27	67.5	17	70.8	0.338
Disagree	24	48.0	19	40.4	13	32.5	7	29.2	
Perceived landscape diversity									
Agree	36	72.0	27	57.5	28	70.0	18	75.0	0.357
Disagree	14	28.0	20	42.5	12	30.0	6	25.0	
Perceived greenery									
Agree	42	84	41	87.2	37	92.5	22	91.7	0.648
Disagree	8	16	6	13.8	3	7.5	2	8.3	
Perceived maintenance									
Agree	36	72.0	35	74.5	31	77.5	19	79.2	0.897
Disagree	14	28.0	12	25.5	9	22.5	5	20.8	
Perceived cleanliness									
Agree	44	88.0	41	87.2	32	80.0	20	83.3	0.704
Disagree	6	12.0	6	12.7	8	20.0	4	16.7	
Perceived crime safety									
Extremely safe	14	28.0	13	27.7	17	42.5	9	37.5	0.624
Quite safe	29	58.0	28	59.6	21	52.5	12	50.0	
Unsafe	7	14.0	6	12.8	2	5.0	3	12.5	
Perceived traffic safety									
Extremely safe	6	12.0	5	10.6	9	22.5	4	16.7	0.176
Quite safe	29	58.0	24	51.1	26	65.0	14	58.3	
Unsafe	15	30.0	18	38.3	5	12.5	6	25.0	

Significant *p*-values are indicated in bold.

**Table 3:** Walk Score between-group comparisons.

Walk Score	Within-Group Change (Intervention)	Within-Group Change (Control)	Between-Group Difference	
	Mean Change (95% CI)	Mean Change (95% CI)	Mean Change (95% CI)	*p*-Value
Average MVPA min per day (*n* = 113)				
0	11.7 (+5.2, +18.8)	−0.8 (−10.4, +8.9)	12.7 (+1.4, +24.0)	**0.028**
1–24	3.5 (−3.6, +10.6)	9.8 (+1.6, +18.1)	−6.3 (−17.0, +4.2)	0.246
25–49	3.7 (−3.5, +11.0)	−1.9 (−10.6, +6.7)	5.7 (−5.5, +16.9)	0.318
≥50	6.3 (−10.8, +23.3)	−3.1 (−10.9, +4.6)	9.4 (−9.1, +28.0)	0.316
% MVPA (*n* = 113)				
0	1.5 (+0.7, +2.3)	−0.1 (−1.2, +1.1)	1.6 (+0.2, +2.9)	**0.023**
1–24	0.4 (−0.5, +1.2)	1.2 (+0.2, +2.1)	−0.8 (−2.1, +0.5)	0.203
25–49	0.5 (−0.4, +1.3)	−0.2 (−1.3, +0.8)	0.7 (−0.6, +2.1)	0.282
≥50	0.6 (−1.4, +2.6)	−0.3 (−1.2, +0.6)	0.9 (−1.3, +3.1)	0.422

Significant *p*-values are indicated in bold.

**Table 4:** Perceived built environment between-group comparisons.

Built Environment Perceptions	Within-Group Change (Intervention)	Within-Group Change (Control)	Between-Group Difference	
	Mean Change (95% CI)	Mean Change (95% CI)	Mean Change (95% CI)	*p*-Value
Perceived close proximity to destinations				
Average MVPA min per day (*n* = 125)				
Agree	4.4 (−2.4, +11.2)	2.6 (−3.8, +9.0)	1.8 (−7.3, +10.9)	0.689
Disagree	7.6 (+1.5, +13.7)	−0.9 (−8.0, +6.1)	8.5 (−0.2, +17.2)	0.055
% MVPA (*n* = 125)				
Agree	0.5 (+1.3, −0.3)	0.3 (−0.4, +1.1)	0.2 (−0.9, +1.2)	0.762
Disagree	0.9 (+0.2, +1.6)	−0.1 (−0.9, +0.7)	1.0 (+0.003, +2.0)	**0.049**
Perceived traffic safety				
Average MVPA min per day (*n* = 125)				
Extremely safe	16.4 (+6.3, +26.5)	1.6 (−8.4, +11.6)	14.8 (+0.9, +28.8)	**0.038**
Quite safe	5.3 (−0.8, +11.6)	1.8 (−4.9, +8.5)	3.5 (−5.3, +12.4)	0.416
Unsafe	3.7 (−4.2, +11.6)	−0.3 (−8.4, +7.7)	4.0 (−7.0, +15.0)	0.464
% MVPA (*n* = 125)				
Extremely safe	1.9 (+0.7, +3.0)	0.3 (−0.9, +1.5)	1.6 (−0.1, +3.2)	0.059
Quite safe	0.7 (−0.04, +1.4)	0.2 (−0.5, +1.0)	0.5 (−0.6, +1.5)	0.376
Unsafe	0.4 (−0.6, +1.3)	−0.04 (−1.0, +0.9)	0.4 (−0.9, +1.7)	0.519

Significant *p*-values are indicated in bold.

## Supplementary Materials

The following are available online at https://www.mdpi.com/1660-4601/16/5/849/s1, Table S1: Bivariate associations between perceived built environment characteristics (gamma coefficients).
