# Supplementary material for: Examining the Associations between Walk Score, Perceived Built Environment, and Physical Activity Behaviors among Women Participating in a Community-Randomized Lifestyle Change Intervention Trial: Strong Hearts, Healthy Communities
Source: Int J Environ Res Public Health. 2019 Mar 8;16(5):849. doi: 10.3390/ijerph16050849 (PMC6427661; doi:10.3390/ijerph16050849)
Supplement: Supplementary file 1 [file ijerph-16-00849-s001.pdf]

Table S1. Bivariate associations between perceived built environment characteristics (gamma coefficients) [a].

Variables	Proximity to Destinations	Sidewalk Availability	Street Shoulder Availability	Bike Lane Availability	Physical Activity Facility Availability	Physical Activity Equipment Availability	Landscape Diversity	Greenery	Maintenance	Cleanliness	Crime Safety	Traffic Safety
Proximity to destinations		**0.405** **	**0.487** **	−0.197	0.156	0.118	−0.072	0.306	0.146	−0.120	0.236	**0.262** *
Sidewalks availability	**0.405** **		**0.656** ***	0.607	**0.471** **	**0.544** ***	0.213	0.165	**0.543** **	**0.482** *	−0.020	0.056
Street shoulder availability	**0.487** **	**0.656** ***		**1.000** **	0.293	**0.371** *	0.302	0.311	**0.460** **	0.139	**0.277** *	**0.482** ***
Bike lane availability	−0.197	0.607	**1.000** **		0.748	0.656	0.558	1.000	0.415	−0.366	0.040	0.320
Physical activity facility availability	0.156	**0.471** **	0.293	0.748		**0.844** ***	**0.601** ***	**0.688** **	**0.352** *	0.132	0.045	0.049
Physical activity equipment availability	0.118	**0.544** ***	**0.371** *	0.656	**0.844** ***		0.240	0.285	**0.357** *	0.242	−0.070	−0.050
Landscape diversity	−0.072	0.213	0.302	0.558	**0.601** ***	0.240		**0.670** *	**0.440** *	0.404	0.079	0.203
Greenery	0.306	0.165	0.311	1.000	**0.688** **	0.285	**0.670** *		−0.040	−0.560	−0.257	−0.216
Maintenance	0.146	**0.543** **	**0.460** **	0.415	**0.352** *	**0.357** *	**0.440** *	−0.040		**0.886** ***	0.162	0.281
Cleanliness	−0.120	**0.482** **	0.139	−0.366	0.132	0.242	0.404	−0.560	**0.886** ***		**0.522** **	**0.551** **
Crime safety	0.236	−0.020	**0.277** *	0.040	0.045	−0.070	0.079	−0.257	0.162	**0.522** **		**0.852** ***
Traffic safety	**0.262** *	0.056	**0.482** ***	0.320	0.049	−0.050	0.203	−0.216	0.281	**0.551** **	**0.852** ***	

[a] Significant p-values are indicated in bold. * $p < 0.05$; ** $p < 0.01$; *** $p < 0.001$.
